# Randomized clinical trial comparing abluminal biodegradable polymer sirolimus-eluting stents with durable polymer sirolimus-eluting stents: Nine months angiographic and 5-year clinical outcomes

**DOI:** 10.1097/MD.0000000000004820

**Published:** 2016-09-23

**Authors:** Haijun Zhang, Xiangfei Wang, Wei Deng, Shenguo Wang, Junbo Ge, Egon Toft

**Affiliations:** a Department of Health Science and Technology, Faculty of Medicine, Aalborg University, Niels, Denmark; b Shanghai Institute of Cardiovascular Diseases, Zhongshan Hospital; c Key Laboratory of Public Health Safety, Ministry of Education, School of Public Health, Fudan University, Shanghai; d Institute of Chemistry, Chinese Academy of Sciences, Beijing, China; e Biomedical Research Center, College of Medicine, Qatar University, Shareh AIJamiaa, Doha, Qatar.

**Keywords:** abluminal biodegradable polymer, late lumen loss, major adverse cardiovascular events, sirolimus-eluting stent

## Abstract

**Background:**

The biodegradable polymer drug-eluting stents (DES) were developed to improve vascular healing. However, further data and longer-term follow-up are needed to confirm safety and efficacy of these stents. This randomized clinical trial aimed to compare safety and efficacy of 2 sirolimus-eluting stents (SES): Cordimax—a novel abluminal biodegradable polymer SES and Cypher Select—a durable polymer SES, at 9 months angiographic and 5-year clinical follow-up.

**Methods:**

We randomized 402 patients with coronary artery disease to percutaneous coronary intervention with Cordimax (n = 202) or Cypher select (n = 200). Angiographic follow-up was performed at 9 months after the index procedure and clinical follow-up annually up to 5 years. The primary endpoint was angiographic in-stent late luminal loss (LLL). Secondary endpoints included angiographic restenosis rate, target vessel revascularization (TVR), and major adverse cardiac events (MACEs; defined as cardiac death, myocardial infarction, or TVR) at 5-year follow-up.

**Results:**

Cordimax was noninferior to Cypher select for in-stent LLL (0.25 ± 0.47 vs 0.18 ± 0.49 mm; *P* = 0.587) and in-stent mean diameter stenosis (22.19 ± 12.21% vs 19.89 ± 10.79%; *P* = 0.064) at 9 months angiographic follow-up. The MACE rates were not different at 1 year (5.9% vs 4.0%, *P* = 0.376); however, MACE rates from 2 to 5 years were lower in the Cordimax group (6.8% vs 13.1%; *P* = 0.039).

**Conclusion:**

Abluminal biodegradable polymer SES is noninferior to durable polymer SES at 9-month angiographic and 1-year clinical follow-up. However, MACE rates from 2 to 5 years were less in the abluminal biodegradable polymer group.

## 1 Introduction

Drug-eluting stents (DES) delivering anti-proliferative drugs from a durable polymer have significantly reduced angiographic and clinical measures of restenosis compared with bare metal stents, with the low risk of adverse events including myocardial infarction (MI) and death.^[1–5]^ However, durable polymers of the first-generation DES have been linked with persistent inflammation, and delayed endothelial healing, which may result in an increased risk of late and very late stent thrombosis (ST).^[6]^ Recent advances in stent technology, including thinner struts and the introduction of biocompatible or biodegradable polymers, have minimized the risk of complications compared with the first-generation DES.^[7–12]^ Several studies have been conducted to test clinical performance of devices with different biodegradable polymers, anti-proliferative drugs, and follow-up duration.^[13–16]^ However, more long-term clinic data are needed to compare biodegradable polymer versus durable polymer sirolimus-eluting stents (SES).

Cordimax stents (Rientech Medical, Shandong, China), which elute sirolimus from abluminal biodegradable polymer, have been shown to have optimized in vitro drug release kinetics and in vivo pharmacokinetics. Animal studies have also shown enhanced endothelialization and vascular healing after stent implantation.^[17]^

The aim of this clinical trial was to evaluate the safety and efficacy of Cordimax abluminal biodegradable SES against Cypher Select durable polymer SES (Cordis Corporation, New Jersey), for 9-month angiographic and 5-year clinical follow-up.

## 2 Methods

### 2.1 Study design and participants

This study is a prospective randomized controlled trial comparing Cordimax and Cypher select stents. Trial enrolled 402 patients with a 1:1 ratio to the 2 study arms. Patients were eligible for enrolment if they were between 18 and 80 years old and intended to undergo percutaneous coronary intervention (PCI) treatment of de novo native coronary artery lesions with a diameter stenosis ≥50% and a reference vessel diameter between 2.5 and 4.0 mm by visual estimation. The major exclusion criteria included acute MI within 1 week, chronic total occlusion, left main coronary artery lesions, bifurcation lesions, and in-stent restenosis. Angiographic follow-up was conducted at 9 months after PCI. Clinical follow-up was performed annually up to 5 years. The trial was approved by the institutional ethics committee. All eligible patients signed written informed consent for participation in the trial.

### 2.2 Study procedure

Patients were treated with standard interventional techniques. Pre-dilation and post-dilation were at the discretion of the treating interventionist. In the event of a bail-out procedure and additional stent requirement, the stent had to be one from the same group as the first implanted stent. All patients received treatment with aspirin (300 mg, at least 24 hours before the intervention) and clopidogrel [loading dose: 300 mg, at least 6 hours before the intervention; for those having taken clopidogrel (75 mg/day) for more than 72 hours, no loading dose was needed]. Anticoagulation with heparin during the procedure was administered according to the protocol recommendations. Dual antiplatelet therapy with aspirin (100 mg/day) and clopidogrel (75 mg/day) was continued for at least 12 months.

### 2.3 Stents

**Figure 1:**
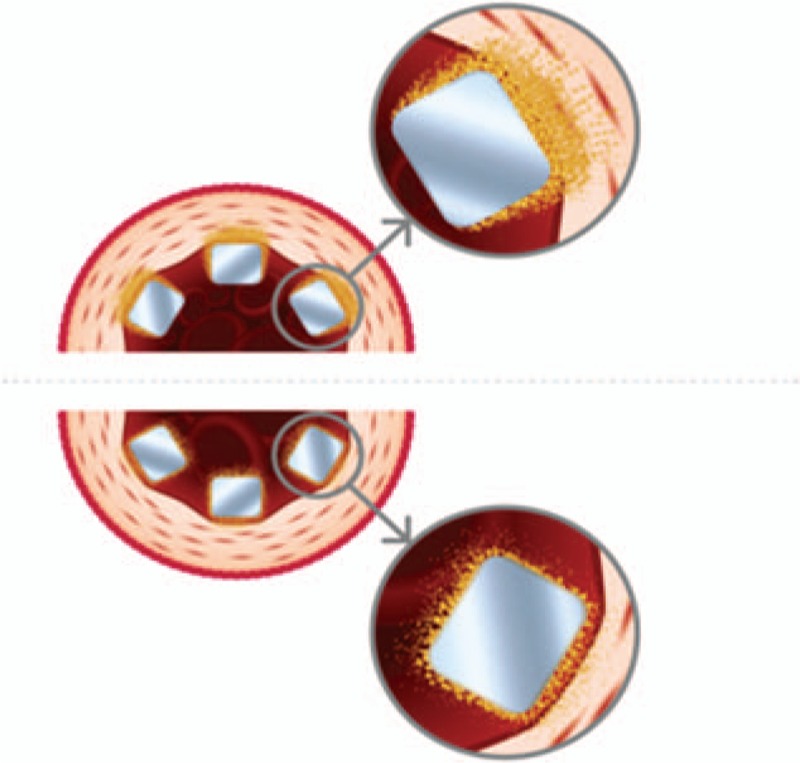
Structure and polymer material of the stent platforms. Top half of the figure depicts abluminal coated stent, whereas the bottom panel shows uniform coating on both luminal and abluminal surfaces.

Cordimax stent has 316L stainless steel platform coated on abluminal surface with a biodegradable polymer polylactide-co-polyglycolide copolymer (75:25 ratio) mixed with anti-proliferative drug sirolimus (Fig. 1). Cypher select is also a 316L stainless steel stent but coated with a durable polymer (Polyethylene-co-vinyl acetate and Poly n-butyl methacrylate). Both stents have similar drug dose (sirolimus 1.4 μg/mm^2^).^[17]^ The Cordimax stent was available in diameters of 2.25, 2.5, 2.75, 3.0, 3.5, and 4.0 mm and in lengths of 9, 12, 14, 16,18, 20, 23, 25, 28, and 33 mm. The Cypher select stents were available in diameters of 2.25, 2.5, 2.75, 3.0, 3.5, and 4.0 mm and in lengths of 12, 15, 18, 23, 28, and 33 mm.

### 2.4 The study endpoints and follow-up

The primary endpoint of the study was in-stent late lumen loss (LLL) at 9 months. Secondary endpoints included device, lesion, and clinical success rates (device success was defined as the attainment of <50% residual stenosis of the target lesion using only the assigned device; lesion success was defined as the attainment of <50% residual stenosis, thrombolysis in myocardial infarction (TIMI) 3 flow, no residual dissection, and thrombosis of the target lesion; clinical success was defined as attainment of lesion success of the target lesion and no in-hospital adverse cardiac events; in-stent and in-segment binary restenosis rates, in-segment LLL, in-stent and in-segment percentage diameter stenosis; MACE, defined as the composite of cardiac death, MI, and target vessel revascularization; and ST—definite and probable ST according to ARC (Academic Research Consortium) definitions (early, late, and very late). MACE and ST were clinically followed up at 1 month, 6 months, 12 months, and annually up to 5 years.^[18]^ Quantitative coronary angiography (QCA) analysis was performed at baseline and 9 months follow-up. All angiograms were evaluated by an independent specialist in core laboratory (Zhongshan Hospital Fudan University, Shanghai) using the QAngio XA Version 7.2 Analysis Software (Medis Medical Imaging System Inc., Leiden, The Netherlands). Standard QCA methodology was used including analysis of the stent and the peri-stent segments of 5 mm proximal and distal to the stent edge. Binary restenosis was defined in every segment (5 mm proximal, 5 mm distal and in-stent) as a >50% diameter stenosis at follow-up. LLL was defined as the difference in minimal lumen diameter (MLD) immediately after stenting and at 9-month follow-up.^[19]^

### 2.5 Statistical analysis

The trial was designed to show noninferiority for in-stent LLL at 9 months. A noninferiority margin was 0.15 mm. The published angiographic results of cypher stent showed an in-stent LLL of 0.15 ± 0.39 mm at 9 months. Assuming an anticipated in-stent LLL of the Cordimax stent of 0.15 mm at 9 months and a noninferiority margin of 0.15 mm, a 2-sided alpha of 0.05% and 90% statistical power would require a minimum number of 288 subjects (144 subjects per group). Assuming a loss to angiographic follow-up rate of 72%, a total sample size of 400 enrolled patients was required. Other treatment group comparisons were performed using the 2-sample *t* test for continuous variables, Fisher exact test for dichotomous variables, and Cochran-Mantel-Haenszel (Modified Ridit scores) for ordinal variables with more than 2 categories. All analyses were performed with the SAS 9.13 software (SAS Institute Inc., Cary, NC).

## 3 Results

### 3.1 Baseline patient and lesion characteristics

In total, 402 patients were randomly assigned 1:1 to treatment with Cordimax stents or Cypher Select stents. A total of 202 patients allocated to Cordimax group received 380 stents and 200 patients allocated to Cypher Select group received 360 stents. All patients were clinically followed up to 5-year follow-up.

Baseline clinical and angiographic characteristics are summarized in Table 1. They were balanced in both groups, including age, gender, and risk factors, including smoking, hypertension, diabetes, previous stroke, previous MI (MI), previous coronary artery bypass grafting (CABG), CCS (Canadian Cardiovascular Society)/Braundward angina pectoris grading, and NYHA (New York Heart Association) functional class. The use of dual antiplatelet therapy was high in both Cordimax and Cypher select groups. The rates of dual antiplatelet therapy were 98.5% versus 98.5% (*P* = 0.99) at 12 months. Baseline angiographic characteristics were also consistent between Cordimax and Cypher Select groups for lesion length, stents diameter, and diameter stenosis pre-PCI (Table 1). Patients in the Cordimax group, compared with Cypher Select group, were more likely to have lesion in right coronary artery (53.5% vs 30%, *P* = 0.001) than left circumflex artery (32.2% vs 20.5%, *P* = 0.008) and received more stents per patient (1.85 ± 1.04 vs 1.56 ± 0.74, *P* = 0.013).

**Table 1:**
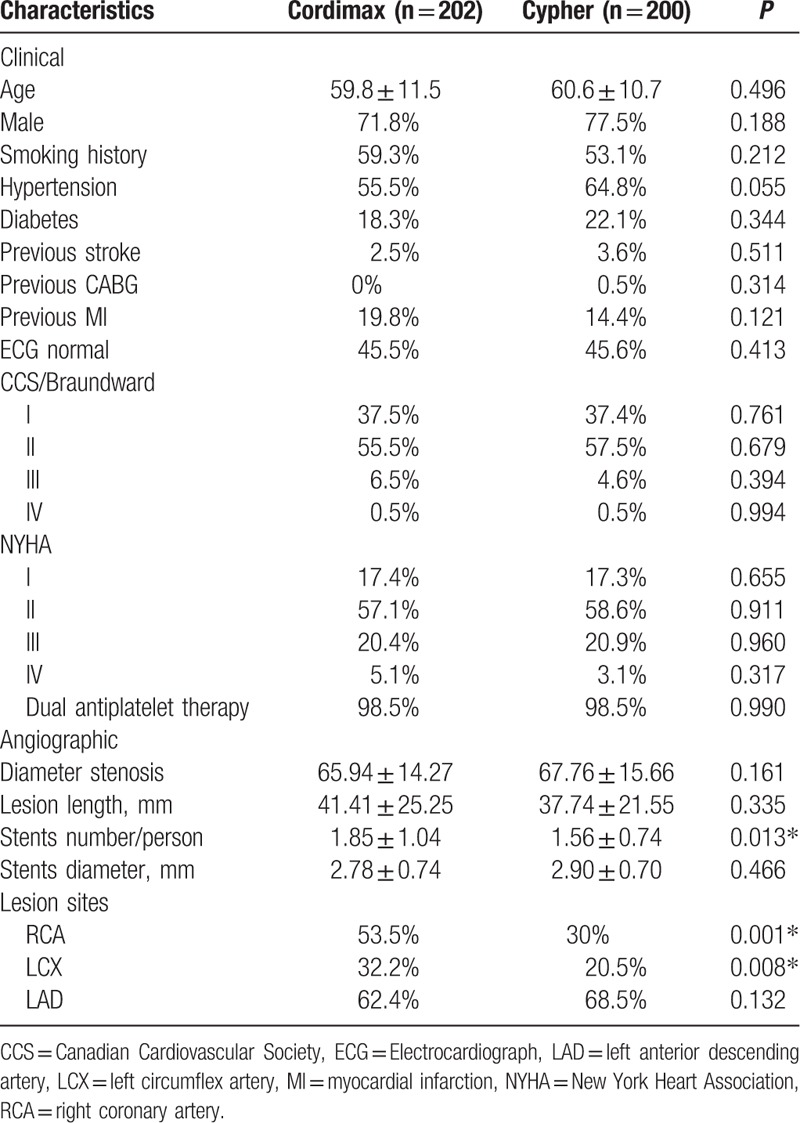
Baseline clinical and angiographic characteristics of patients.

### 3.2 Angiographic outcomes

Angiographic follow-up at 9 months was completed in 299 (74.4%) patients. A total of 145 patients (71.8%) allocated to Cordimax group and 154 patients (77.0%) allocated to Cypher select underwent follow-up angiography. Device success (100% vs 100%, *P* = 1.0) and lesion success (99.98% vs 98.74%, *P* = 1.0) was high and no different between the 2 groups. According to the pre-specified noninferiority margin, Cordimax stents were noninferior to Cypher Select for the primary angiographic end point of in-stent LLL at 9 months [0.25 ± 0.47 vs 0.18 ± 0.49 mm; difference = 0.07 ± 0.48 mm; 95% confidence interval (95% CI) -0.02 to 0.17]. Other angiographic outcomes including percent in-stents restenosis, percent segment restenosis, and minimum lumen diameter of target vessel were also not different between the 2 groups (Table 2).

**Table 2:**
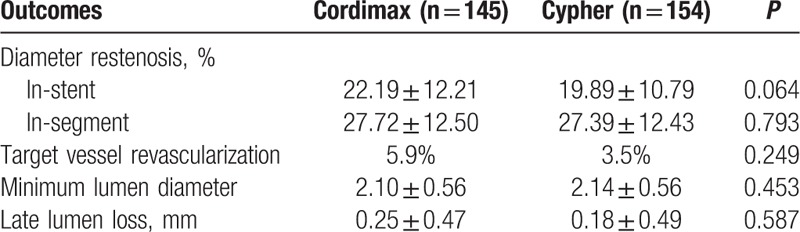
Angiographic outcomes at 9-month follow-up.

### 3.3 Clinical outcomes

**Figure 2:**
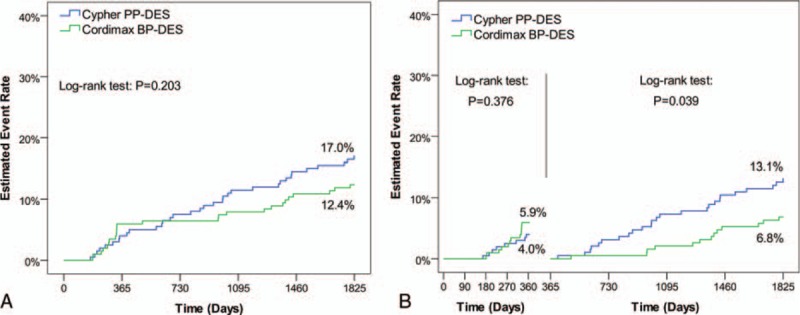
Kaplan-Meier estimates of primary endpoint until 5-year follow-up. BP = biodegradable polymer, DES = drug-eluting stent, PP = permanent polymer.

There were no deaths in Cordimax group and 1 death in Cypher select group (noncardiovascular cause) during first year. Clinical event rates during the 5 years follow-up are summarized in Table 3. The cumulative rate of MACE, TVR, cardiac death, and MI were comparable for patients in Cordimax and Cypher Select groups (Table 3). However, it was found that the 2 groups had significant difference in the cumulative pursue rate of MACE and TVR from 2 to 5 years (cumulative pursue rate = annual cumulative rate - 1st year rate) (Table 3 and Fig. 2). The cumulative pursue rates of MACE and TVR in Cordimax were lower than those of Cypher Select (Fig. 2). There was no ARC-defined definite and probable ST in the Cordimax group. One case was proved to be definite very late ST in Cypher Select group (Table 3).

**Table 3:**
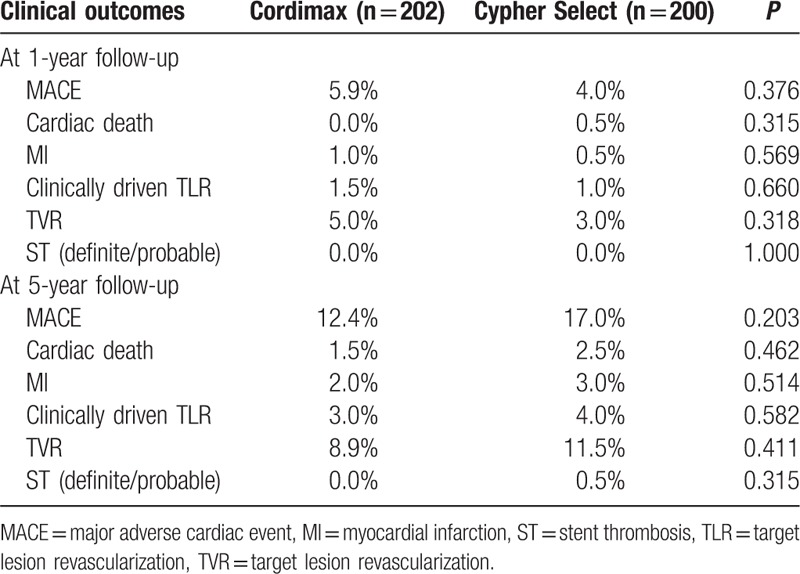
Clinical outcomes up to 5 years follow-up.

## 4 Discussion

Our study has shown that abluminal biodegradable polymer SES was noninferior to durable polymer SES for angiographic in-stent LLL at 9-month and MACE at 1-year follow-up. However, the events rate in the subsequent years tended to be lower in the abluminal biodegradable polymer group.

Other studies have also shown noninferiority of biodegradable polymer based DES for angiographic outcomes at 9 to 12 months follow-up. Our results corroborate with data from LEADERS,^[7,10]^ ISAR-TEST,^[9]^ CREATE,^[8]^ EVOLVE,^[20]^ and BIOFLOW trials.^[21]^ The results therefore confirm the efficacy of biodegradable polymer DESs against the first-generation permanent-polymer DES. The abluminal biodegradable polymer SES as compared with durable polymer SES tended to reduce annual cumulative rate of MACE, TVR, cardiac death, MI, and ST than durable polymer SES in 5 years follow-up. However, the differences were not statistically significant (*P* > 0.05). Nevertheless, abluminal biodegradable polymer SES showed superiority in longer term safety, by reducing cumulative pursue rates of MACE, TVR, and ST in 5 years. Cordimax stent has polylactide-co-polyglycolide copolymer (75:25 ratio), which degrades into carbon dioxide and water after the drug (sirolimus) is released.^[17]^ These polymers and their degradation products are more biocompatible and less prone to hypersensitivity reactions. Although our study was not powered for clinical outcomes, our results are consistent with other studies and a meta-analysis suggesting better long-term outcomes with biodegradable polymer DES. ^[6,9,13]^

Our study had relatively lower incidence of MACE, TVR, and ST in both groups. This may partly reflect inclusion of relatively lower risk patients as the major exclusion criteria included acute MI within 1 week, chronic total occlusion, left main coronary artery lesions, bifurcation lesions, and in-stent restenosis, which is different from some other studies.^[7,9,10,13,16,20–23]^. Furthermore, adherence to optimal medical therapy, especially DAPT (which was 98% at 1 year), is higher than other trials.^[24]^

New generation DES have been developed to improve device performance and safety, with a specific attention at reducing delayed re-endothelialization and very late ST. Cordimax stent employs patented technique of the asymmetric coating, smearing, and modulating drug release, which would be beneficial for the vascular endothelium to regenerate.^[17]^ The abluminal polymers tend to effectively prevent the vascular restenosis and decrease the incidence of late thrombosis, which contribute to reduction in longer-term adverse events. Further larger scale trials of this device, powered for clinical outcomes, are warranted.

### 4.1 Limitations

This study has several limitations. First, the sample size was calculated to assess a difference in LLL, which is a surrogate endpoint for clinical restenosis. Second, patients included in the study were selected strictly by angiographic and clinical characteristics rather than an all-comers set-up; thus, the results may not be applicable to all patient groups. Finally, the present study did not include additional intracoronary imaging guidance (such as intravascular ultrasound, optical coherence tomography).

## 5 Conclusion

Compared with durable polymer Cypher Select, abluminal biodegradable polymer Cordimax stents showed similar efficacy for the primary end point in stent LLL and potentially better longer term safety for MACE, TVR, and ST.
